# The transcriptional regulator CprK detects chlorination by combining direct and indirect readout mechanisms

**DOI:** 10.1098/rstb.2012.0323

**Published:** 2013-04-19

**Authors:** Laura R. Kemp, Mark S. Dunstan, Karl Fisher, Jim Warwicker, David Leys

**Affiliations:** Faculty of Life Sciences, Manchester Institute of Biotechnology, University of Manchester, 131 Princess Street, Manchester M1 7DN, UK

**Keywords:** CprK, organohalide respiration, transcriptional regulation, phenol p*K*_a_, green fluorescent protein, halogen detection

## Abstract

The transcriptional regulator CprK controls the expression of the reductive dehalogenase CprA in organohalide-respiring bacteria. *Desulfitobacterium hafniense* CprA catalyses the reductive dechlorination of the terminal electron acceptor *o*-chlorophenol acetic acid, generating the phenol acetic acid product. It has been shown that CprK has ability to distinguish between the chlorinated CprA substrate and the de-halogenated end product, with an estimated an estimated 10^4^-fold difference in affinity. Using a green fluorescent protein GFP_UV_-based transcriptional reporter system, we establish that CprK can sense *o*-chlorophenol acetic acid at the nanomolar level, whereas phenol acetic acid leads to transcriptional activation only when approaching micromolar levels. A structure–activity relationship study, using a range of *o*-chlorophenol acetic-acid-related compounds and key CprK mutants, combined with p*K*_a_ calculations on the effector binding site, suggests that the sensitive detection of chlorination is achieved through a combination of direct and indirect readout mechanisms. Both the physical presence of the bulky chloride substituent as well as the accompanying electronic effects lowering the inherent phenol p*K*_a_ are required for high affinity. Indeed, transcriptional activation by CprK appears strictly dependent on establishing a phenolate–K133 salt bridge interaction, rather than on the presence of a halogen atom *per se*. As K133 is strictly conserved within the CprK family, our data suggest that physiological function and future applications in biosensing are probably restricted to phenolic compounds.

## 1. Introduction

While halide chemistry is a mainstay of modern chemical synthesis and products, the presence of halide atoms within biological molecules is a relatively rare occurrence [1,2]. Indeed, the presence of halide atoms in xenobiotic molecules often renders them recalcitrant to biological mineralization and can cause or augment toxic effects. The high toxicity, persistence and bioaccumulation of chlorinated molecules such as polychlorinated biphenyls (PCBs) or dioxins is of particular concern, and pollution of the environment and/or food chain by these compounds is often exacerbated by detection problems [3,4]. Organohalide-respiring bacteria have been shown to use a range of chlorinated molecules as terminal electron acceptors, and these organisms as well as the molecular components underpinning this process have the potential for future applications in bioremediation/biosensing of PCB/dioxin-type compounds [5]. Desulfitobacteria are strictly anaerobic, low-G + C bacteria, belonging to the Firmicutes, Clostridia and Peptococcaceae [6] and have been studied for over a decade because of their ability to dehalogenate organic compounds via organohalide respiration. The bacterium *Desulfitobacterium hafniense* DCB-2 is able to dechlorinate many chlorinated compounds including: pentachlorophenol (PCP), tetrachloroethene and 3-chloro-4-hydroxyphenylacetic acid (OCPA) [7,8]. The key enzymes that catalyse the final reduction step are known as reductive dehalogenases (Rdhs). Rdh enzymes are predicted to be membrane anchored by a small protein and are corrinoid/iron–sulfur-containing proteins. CprA Rdh isolated from *Desulfitobacterium* species are able to dechlorinate a range of halogen*-*substituted phenolic compounds [9–11]. In *D. dehalogenans* and *D. hafniense*, the CprA genes are under tight transcriptional control by the regulator CprK [9]. CprK belongs to the CRP/FNR family of regulators and, following binding to the effector *o*-chlorophenol acetic acid, binds a specific DNA promoter sequence within the *cpr* gene cluster called a dehalobox (TTAAT–N_4_–ATTAA) [12–17].

The CRP/FNR family of transcription regulators is ubiquitous in bacteria and is able to respond to a wide spectrum of signals from within the cell and its environment [18,19]. All members of this superfamily contain an N-terminal sensor module able to bind the effector—OCPA in the case of CprK—or undergo effector-mediated modification. Allosteric effects of ligand binding or modification of the N-terminal sensor domain is transmitted to the C-terminal DNA-binding domain through the central pair of α-helices that connect both domains and form a large part of the dimer interface. The exact allosteric networks that underpin the regulator function are distinct for each of those family members that have been studied in molecular detail (i.e. CooA, CprK, CRP) reflecting the versatility in the type of signal/ligand sensed (respectively CO gas, cAMP, halogenated phenolic compounds; for reviews see [18,19]). Furthermore, the dimeric nature of the CRP/FNR family members appears to be exploited differently depending on the type of signal sensed. In the case of CRP, mild negative cooperativity between both binding sites is hypothesized to allow the molecule to report on a wide range of cAMP concentrations, as both the half and fully occupied dimers (each with distinct DNA-binding affinities) are populated to significant levels [20]. By contrast, it has been proposed that CprK exhibits either extreme positive or no cooperativity, both effectively leading to an equilibrium between unbound and fully bound CprK [16]. In the latter case, this presumably reflects the need for CprK to function as a single threshold on–off switch.

**Figure 1.**
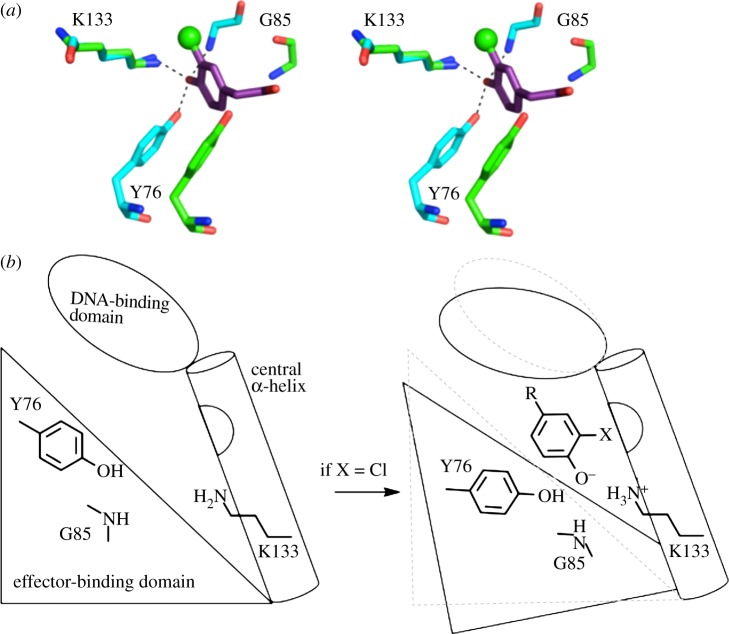
(*a*) Stereo view of an overlay of the CprK effector binding site in the OCPA-bound (with cyan carbon atoms) and unbound (green carbon atoms) conformations. The OCPA chloride atom is shown as a green sphere, and the remainder of the effector molecule as atom coloured sticks (purple carbon atoms). (*b*) Schematic of the proposed CprK mechanism. CprK is presented as a single monomer for clarity; for a more detailed schematic that includes the proposed cooperativity between CprK monomers, see Joyce *et al*. [14].

CprK displays remarkable ability to distinguish between the halogenated OCPA and the corresponding non-halogenated 4-hydroxyphenylacetic acid (HPA), estimated from indirect *in vitro* measurements at 10^4^ difference in affinity [13–16]. This not only ensures the reductive dehalogenase is only produced when substrate is present, but also avoids an accumulation of the product from the dehalogenation reaction leading to persistent signalling. Detailed structural information on CprK in a range of states has revealed that OCPA binds to the effector domain central α-helix pair interface, with concomitant rigid body reorientation of the effector domain with only minor changes in residues lining the ligand-binding site. The chloride atom is located in a hydrophobic pocket at the central α-helix pair surface (formed by Y130, L131, K133, V134). No directional halogen bonding contacts can be observed [21]. The relatively weak nature of these hydrophobic contacts and the fact these residues only undergo minor changes upon ligand binding appears inconsistent with the marked difference in affinity and response displayed by CprK towards OCPA and HPA. We proposed that in addition to detecting the presence of the halogen atom directly, through hydrophobic contacts, CprK senses the p*K*_a_ of the phenolic moiety [14,16]. The phenol group is within hydrogen bonding distance of Y76, the backbone NH of G85 and the conserved K133, with a potential salt bridge forming between the deprotonated phenolate group and K133 (figure 1). The nature of this network would depend on the p*K*_a_ of the phenolic moiety, which is influenced by the presence of the halogen atom. In this study, we describe the *in vivo* transcriptional activation levels of wild-type (WT) CprK and a number of selected mutants for a range of OCPA-like effector molecules, combined with calculations of the K133/phenol protonation states within the crystal structures. Our data reveal a pivotal role for K133 and establish a link between phenol p*K*_a_ and effector affinity. We furthermore reveal that the presence of a phenol group, rather than the halogen substituent itself, is the key prerequisite for transcriptional activation by CprK.

## 2. Results and discussion

### (a) Development of a GFP_UV_-based effector reporter assay

**Figure 2.**
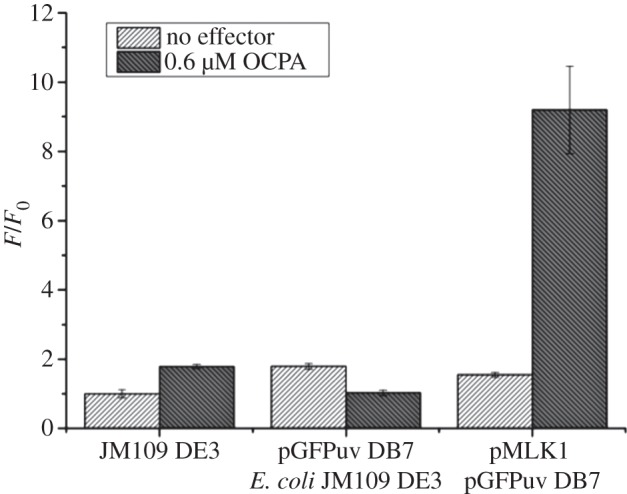
GFP_UV_-DB7 reporter assay. Ratio of fluorescence levels at *t* = 3 h/*t* = 0 in the presence and absence of 0.6 μM OCPA obtained for *E. coli* JM109 DE3 cells, containing either no plasmid, pGFP_UV_-DB7 only or co-transformed with pGFP_UV_-DB7 and pMLK1. Fluorescence values are normalized to cell density levels. Error bars indicate the standard deviation of three separate readings.

**Figure 3.**
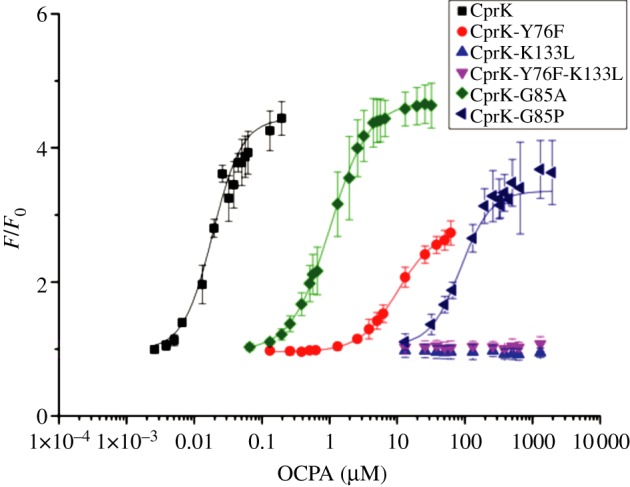
Fluorescence levels are depend on OCPA concentration. The ratio of fluorescence of pGFP_UV_-DB7 *E.coli* JM109 DE3 cells expressing either CprK or selected mutant versions, incubated at various OCPA concentrations for 3 h to the fluorescence obtained at *t* = 0. Fluorescence values are normalized to cell density levels. Error bars indicate the standard deviation of three separate readings.

Previous studies have shown that OCPA-dependent transcriptional activation by CprK can be monitored in *Escherichia coli* [17,22]. To avoid any effects coupled to the redox state of the cell, we used a CprK C200S mutant as the basis for all our studies (referred to hereafter as CprK), as it was previously shown that oxidative disulfide bond formation involving C200 inactivates the regulator [23]. A region comprising 123 nucleotides of the upstream dehalobox promotor region of CprA1, containing CprK binding element dehalobox 7 (DB7), was cloned into pGFP_UV_ in order to create a novel *in vivo* reporter system that makes use of an optimized variant of the green fluorescent protein (GFP_UV_) from *Aequorea victoria*. Fluorescence levels of the pGFP_UV_-DB7 containing *E. coli* cells were comparable to non-transformed cell-lines indicating minimal basal transcription of GFP (figure 2). No significant increase in fluorescence levels could be detected in the presence of OCPA or similar compounds. Fluorescence levels for cells containing both pGFPuv-DB7 and pET28a-CprK were comparable to the pGFP_UV_-DB7 single transformants, indicating negligible levels of transcriptional activation occur in the absence of the halogenated effector. By contrast, addition of 0.6 μM OCPA to the media leads to a nearly 10-fold increase in fluorescence levels, indicating CprK-mediated, OCPA-dependent transcriptional activation of the *GFP_UV_* gene occurs within the *E. coli* host. Although a comparison with published β-galactosidase-based assays reveals the amplification level achieved using GPF_UV_ to be significantly smaller (over a 20-fold increase has been reported [17,22]), the pGFP_UV_-DB7 system allows for a more accurate and direct *in vivo* observation of protein expression levels. We measured fluorescence levels as a function of OCPA concentration in the medium and found a sigmoidal dependence in the data that could be fitted to a Hill equation, with a Hill coefficient of *n* = 2.0 ± 0.2 and an apparent *K*_d_ of 0.019 ± 0.002 μM (figure 3 and table 1). Our apparent *K*_d_ is the binding constant calculated as the concentration at which effector induces half *F*_max_ (maximum fluorescence). Our apparent *K*_d_ data are significantly smaller than that previously reported *in vitro* for OCPA (*K*_d_ = 0.8–3.5 μM) [13–16]. In contrast to the direct measurement of formation of the CprK : OCPA complex for the *in vitro* experiment, the *in vivo* data probably report on the formation of the (CprK : OCPA : dehalobox : RNA polymerase) quaternary complex. Indeed, it was found that the CprK affinity for OCPA was increased approximately 10-fold when in the presence of a DNA promoter fragment [13]. It appears that under the conditions used for the pGFP_UV_-DB7-based system, the apparent *K*_d_ for OCPA is approximately 100-fold smaller than the established *in vivo K*_d_ values. Furthermore, the *in vivo* data could only be accurately modelled using a Hill equation, revealing positive cooperative behaviour that has not previously been observed for *in vitro* experiments. It is important to keep in mind that transport processes could affect the intracellular OCPA concentrations across the membrane, although it would seem logical to assume this would affect OCPA and OCPA-like components in a similar manner. To confirm the apparent *K*_d_ value observed reflects inherent CprK affinity for the effector molecule, we measured the increase in fluorescence levels for a range of aromatic compounds. The relationship between the nature of the aromatic compounds tested and the apparent *K*_d_ obtained strongly suggests that these experiments directly probe inherent CprK affinity.

**Table 1.** Apparent *in vivo K*_d_ for OCPA determined for CprK and selected mutant forms.

CprK	*K*_d_ (apparent; μM)	*n*	adj. *R*^2^
CprK	0.019 ± 0.002	2.0 ± 0.2	0.988
K133L	no response	n.a.	n.a.
Y76F	10.2 ± 0.9	1.6 ± 0.1	0.996
G85A	1.03 ± 0.06	1.5 ± 0.1	0.998
G85P	86.7 ± 5.8	1.9 ± 0.3	0.995

### (b) Substitution of the OCPA *ortho*-functional group affects *in vivo* transcriptional activation by CprK

**Figure 4.**
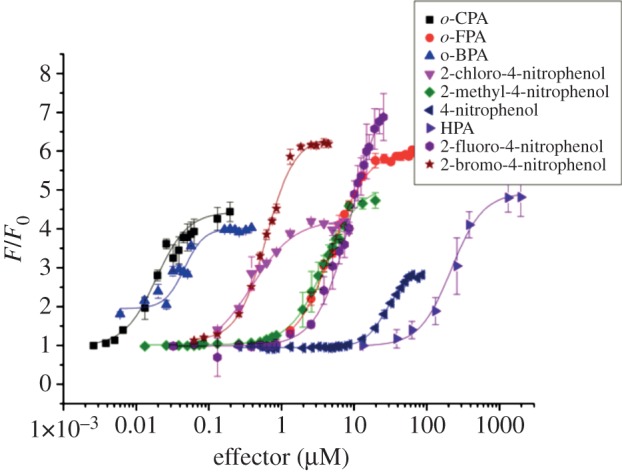
Fluorescence levels depend on the nature of the phenolic effector. The ratio of fluorescence of pGFP_UV_-DB7 *E. coli* JM109 DE3 cells expressing WT CprK1, incubated at various concentrations of a phenolic effector for 3 h to the fluorescence obtained at *t* = 0. Fluorescence values are normalized to cell density levels. Error bars indicate the standard deviation of three separate readings.

Figure 4 and table 2 show apparent *K*_d_ values and associated Hill constants for a range of 4-hydroxyphenylacetic acid molecules with different *o*-substituents (Br, Cl, F, H). The relative difference in atomic radius of the four distinct *o*-substituents can only partly explain the observed trend in associated *K*_d_ values. There is an approximately 10 000-fold difference in *K*_d_ between OCPA and HPA, according to previously determined, albeit indirect, estimations of the discriminatory power of CprK for the substrate and product [14,16]. The atomic radius of the *o*-substituent clearly plays a role in this mechanism, but is not the only determinant. In this respect, the 40-fold difference between apparent *K*_d_ values for HPA and *o*-3-fluoro-4-hydroxyphenyl acetic acid (*o*-FPA) is noteworthy, as H and F have a very similar atomic radius but have distinct electronegativity. This supports the p*K*_a_ interrogation hypothesis for the mechanism of CprK, as the p*K*_a_ between HPA and *o*-FPA is affected by the F-substituent. To explore the relationship between atomic radius of the PA *o*-functional group and CprK affinity in the absence of significant p*K*_a_ effects, we tested a range of *p*-nitrophenolic compounds. In this series, the electron-withdrawing effects of the *p*-nitro substituent largely determine the phenol p*K*_a_.

**Table 2.** Apparent *in vivo* *K*_d_ for a range of aromatic compounds for CprK. For general structure of effectors, see figure 5.

effector	R_1_ (p*K*_a_ value)	R_2_	R_3_	apparent *K*_d_ (μM)	*n*	adj. *R*^2^
4-hydroxyphenylacetic acid (HPA)	–OH (10.2)	–H	–CH_2_–COOH	210 ± 17	2.1 ± 0.2	0.995
3-fluoro-4-hydroxyphenylacetic acid	–OH (8.8)	–F	–CH_2_–COOH	4.96 ± 0.26	1.8 ± 0.2	0.998
3-chloro-4-hydroxyphenylacetic acid (OCPA)	–OH (8.5)	–Cl	–CH_2_–COOH	0.019 ± 0.002	2.0 ± 0.2	0.988
3-bromo-4-hydroxyphenylacetic acid	–OH (8.5)	–Br	–CH_2_–COOH	0.046 ± 0.003	3.2 ± 0.5	0.967
4-nitrophenol	–OH (7.2)	–H	–NO_2_	27.7 ± 1.5	2.8 ± 0.2	0.993
2-fluoro-4-nitrophenol	–OH (5.7)	–F	–NO_2_	10.0 ± 1.2	4.7 ± 0.6	0.998
2-chloro-4-nitrophenol	–OH (5.5)	–Cl	–NO_2_	0.402 ± 0.050	1.7 ± 0.2	0.999
2-bromo-4-nitrophenol	–OH (5.5)	–Br	–NO_2_	0.613 ± 0.016	2.1 ± 0.1	0.999
2-methyl-4-nitrophenol	–OH (7.4)	–CH_3_	–NO_2_	3.92 ± 0.33	1.8 ± 0.1	0.993
4-nitroaniline	–NH_2_	–H	–NO_2_	no binding	n.a.	n.a.
2-chloro-4-nitroaniline	–NH_2_	–Cl	–NO_2_	no binding	n.a.	n.a.
2-methyl-4-nitroaniline	–NH_2_	–CH_3_	–NO_2_	no binding	n.a.	n.a.
3-chloro-phenylacetic acid	–H	–Cl	–CH_2_–COOH	no binding	n.a.	n.a.

### (c) Both atomic radius and electronegativity of the phenylacetic acid *ortho*-functional group determine CprK affinity

Table 2 and figure 4 show apparent *K*_d_ values and associated Hill constants for a range of *p*-nitrophenolic compounds (CH_3_, Br, Cl, F and H as *o*-substituent). Unlike the PA series, the p*K*_a_ of the *p*-nitrophenol series is largely determined by the nitro-group. A comparison between OCPA and the corresponding *o*-chloro-*p*-nitrophenol reveals approximately a 20-fold difference in affinity, probably owing to the loss of hydrogen bonding interactions with the acetic acid moiety. Within the *p*-nitrophenol series, there is a clear trend between atomic radius of the *o*-substituent and the corresponding apparent *K*_d_. An approximately sevenfold difference between *o*-methyl-*p*-nitrophenol and *p*-nitrophenol reveals that atomic radius plays a significant part in the CprK mechanism, as both C and H have very similar electronegativity values. These data support the hypothesis that CprK determines the nature of the *o*-substituent through both direct effects (atomic radius through van der Waals interactions) and indirect effects (hydrogen bonding network and salt bridge to the phenolate moiety). To investigate whether CprK has the ability to function with non-phenolic effector molecules, we investigated the effects of *o*-chloro-*p*-nitroaniline and of 3-chloro-phenylacetic acid on fluorescence levels.

### (d) Only phenolic compounds elicit transcriptional activation by CprK

No increase in fluorescence levels could be observed for a range of aniline compounds such as *o*-chloro-*p*-nitroaniline, or 3-chloro-phenylacetic acid, a molecule highly similar to OCPA but lacking the phenolic group (table 2). It is interesting to note that the *D. hafniense* strain PCP-1 is able to dechlorinate pentachloroaniline and 2,3,5,6-tetrachloroaniline, but only after induction by 3,5-dichlorophenol and 2,4,6-trichlorophenol [24]. In the case of 3-chloro-phenylacetic acid, a lack of apparent binding can easily be explained by the loss of the hydrogen bonding network and the K133 salt bridge to the phenolate moiety. Furthermore, the lack of any effect on fluorescence levels for *o*-chloro-*p*-nitroaniline suggests this polar network of interactions cannot be established with an –NH_2_ group. In this respect, the relative difference between –NH_2_ and –OH (phenol) or –O^−^ (phenolate), respectively, would support the hypothesis that CprK binds only to the phenolate form. The phenolate group is within hydrogen bonding distance of Y76, backbone NH of G85 and K133 (figure 1), putatively receiving a hydrogen bond from each amino acid and establishing a salt bridge with the conserved K133. The fact that the latter amino acid is strictly conserved in CprK paralogues suggests a common role in their recognition of (halogenated) phenolic effectors. By contrast, Y76 is not conserved, and a previous study has revealed a Y76F mutation leads to an approximately 10-fold decrease in the *K*_d_ for OCPA, consistent with the interruption of a single hydrogen bond [14]. To further probe the relative contributions made by each amino acid to the phenolate-binding polar network, we studied the properties of Y76F, G85A, G85P and K133L CprK mutants.

### (e) The conserved K133 is crucial to CprK functionality

Table 1 and figure 3 show the dependence of fluorescence levels on OCPA concentrations for Y76F, G85A, G85P and K133L CprK mutants. Western blot analysis of the soluble cell fractions transformed with CprK WT or mutant expression plasmids reveals similar levels of soluble CprK protein in the cell (data not shown). Compared with the apparent *K*_d_ of WT CprK (0.019 ± 0.002 μM) for OCPA, the Y76F mutant has an approximately 500-fold decreased affinity for OCPA. The G85P mutation leads to an even more dramatic 4500-fold decrease, but this is probably not only a consequence of removal of the backbone NH hydrogen bond, but also because of the increased size and restricted conformational freedom of the proline side chain. In this respect, the more conservative mutation G85A, which does not remove the possibility of a hydrogen bond between the phenolate and position 85 and reveals the extra presence of a methyl group, already significantly affects OCPA affinity, leading to an approximately 50-fold activity decrease. While mutations at position 76 or 85 retain some activity, mutation at position 133 leads to a complete loss of transcriptional activation response. This clearly establishes K133 as the key contributor to the polar network established with the phenol moiety.

### (f) p*K*_a_ calculations for the CprK effector binding site

We used computational modelling to provide further insight into CprK effector binding by modelling p*K*_a_ and protonation values for K133 and the OCPA or HPA phenolic group, respectively. We used available crystal structures to model a K133L mutant structure, as well as a putative complex of the WT protein with HPA. Table 3 shows the average protonation state of K133 and the OCPA phenolic group, averaged over both effector sites within the CprK dimer, at pH 7.5 for the different CprK complexes (i.e. 1 = protonated, 0 = deprotonated). The most striking feature of calculated protonations for effector sites is that K133 and phenol protonations sum to 1. These titratable sites are adjacent within a relatively low dielectric environment, shielded from bulk water, leading to strong interaction and tight coupling. However, the strong interaction between protonated K133 and the deprotonated phenolate is largely balanced by the desolvation penalties associated with these ionizations. As a result, the location of the shared proton is predicted to shift from K133 to phenol according to the phenol p*K*_a_. Thus, for OCPA complexes, the balance lies towards the ion pair, whereas for a putative HPA–CprK complex, the balance is predicted to be towards the neutral pair. In the absence of the ligand, but within the ligand-bound protein conformation context, K133 is predicted to be unprotonated. For a modelled K133L mutation, the phenol group of the bound effector is predicted to be protonated, emphasizing the tight coupling of K133 and phenol groups. These calculations are consistent with the p*K*_a_ readout hypothesis for effector binding to CprK. The effector binding site is poised, in terms of K133/phenol charge distribution, and we estimate a contribution of around 5 kJ per mole more favourable for a single OCPA than a single HPA, at pH 7.5.

**Table 3.** Average protonation state of K133 and the OCPA/HPA phenolic group, averaged over both effector sites within the CprK dimer, at pH 7.5 (i.e. 1 denotes protonated; 0 denotes deprotonated).

CprK structure	K133 protonation	phenol protonation	K133 + phenol protonation
apo-structure (PDB code 3e5q)	0.7	n.a.	n.a.
binary OCPA complex (3e5x)	0.9	0.1	1.0
ternary OCPA and DNA complex (3e6c)	0.8	0.2	1.0
3e5x-derived model binary HPA complex	0.4	0.6	1.0
3e6c-derived model for ternary HPA and DNA complex	0	1.0	1.0
3e5x-based binary OCPA K133L model	n.a.	1.0	1.0
3e5x-derived apo-structure (in binary complex conformation)	0	n.a.	n.a.

## 3. Conclusions

Since their discovery a few decades ago, organohalide-respiring bacteria have been studied to determine the biochemical basis for their unusual metabolism, recently culminating in the genome sequence of a range of model species [25,26]. The unusual nature of the substrate, a halogenated molecule, presents these organisms with new challenges for which they have found corresponding novel biochemical solutions [5]. This, combined with the potential for a future application in bioremediation/biosensing of halogenated xenobiotics of these organisms, warrants a detailed investigation of the organohalide respiratory process. Although a range of transcriptional regulators have been implicated in the organohalide respiration process, reflecting the wide range of compounds used as electron acceptors, CprK is the only protein for which a wealth of biochemical data are available. This transcriptional regulator has the ability to distinguish between the chlorinated effector and the corresponding non-halogenated component by an approximately 10 000-fold difference in affinity. Our *in vivo* measurement of apparent *K*_d_ establishes that CprK has the potential to sense halogenated molecules at nanomolar levels, and directly confirms the striking difference between OCPA and HPA affinity. Furthermore, by studying the effects of substitution of key CprK amino acids or OCPA substituents, we have been able to confirm that this is due to a combination of both direct and indirect readout mechanisms. While the physical presence of the halogen substituent contributes to affinity via van der Waals interactions, the main detection mechanism appears indirect: sensing the effects of the electronegative halogen substituent on the phenol p*K*_a_. Indeed, transcriptional activation by CprK is entirely dependent on the presence of K133 and a phenolic effector molecule, rather than the strict presence of a halogen atom *per se*. p*K*_a_ calculations of the CprK effector binding site indeed support the proposal that an ionic interaction is formed between K133 and the effector. The fact that K133 is strictly conserved within the CprK family suggests its function is limited to sensing the presence of halogenated phenolic components in the environment. We would thus predict that application of CprK family members or variants in biosensing is likely to remain limited to phenolic compounds.

## 4. Material and methods

### (a) Strains, plasmids and growth conditions

Characteristics of the strains and plasmids used in this study are listed in table 4. *Escherichia coli* DH5α cells were used as a host for mutagenized plasmids. *Escherichia coli* JM109 DE3 cells were used for co-transformations and GFP assay. Origins of replication of co-transformed plasmids were within the same incompatibility group (table 4), but in possession of different selective markers. It has been shown that it is possible to maintain high-copy vectors from the same incompatibility group, with differing selective markers in *E. coli* over the course of 5 days [28]. In order to maximize plasmid survival, cells were co-transformed, and assays completed within 5 days. Kanamycin (30 μg ml^−1^) was used as a selective agent for transformations of mutagenized plasmids. Ampicillin (100 μg ml^−1^) was used as a selective agent for cloning of pGFP_UV_-DB7. Kanamycin (30 μg ml^−1^) and ampicillin (100 μg ml^−1^) were used as selective agents for co-transformation of CprK-derived and GFP-containing plasmids. CprK-derived genes were under the basal control of a T7 promoter, in the absence of isopropyl β-d-1-thiogalactopyranoside. Cells were grown in LB broth or agar at 37°C.

**Table 4.** Strains and plasmids used in this study.

strain/ or plasmid	relevant characteristics	origin of replication	promoter	reference
*E. coli*				
DH5α	*fhuA2* *Δ*(*argF-lacZ*)*U169 phoA glnV44* *Φ80Δ* (*lacZ*)M15 *gyrA96 recA1 relA1 endA1 thi-1 hsdR17*			New England Biolabs
JM109 (DE3)	*end*A1, *rec*A1, *gyr*A96, *thi, hsd*R17 (r_k_^–^, m_k_^+^), *rel*A1, *sup*E44, λ–, *Δ*(*lac-pro*AB), [F′, *tra*D36, *pro*AB, *lac*I^q^Z*Δ*M15], lDE3			Promega
pET28a(+)	Km^R^, pET28a(+)	f1, ColE1	T7	Novagen
pMLK1	*D. hafniense cprK_C200S_* cloned into pET28a	f1, ColE1	T7	this study
pMLK2	*D. hafniense cprK_C200S-K133L_* cloned into pET28a	f1, ColE1	T7	this study
pMLK3	*D. hafniense cprK_C200S-Y76F_* cloned into pET28a	f1, ColE1	T7	this study
pMLK4	*D. hafniense cprK_C200S-G85A_* cloned into pET28a	f1, ColE1	T7	this study
pMLK5	*D. hafniense cprK_C200S-G85P_* cloned into pET28a	f1, ColE1	T7	This study
pGFP_UV_	Am^R^, pGFP_UV_	pUC	lac	[27]
pGFP_UV_-DB7	dehalobox promoter cloned into pGFP_UV_	pUC	DB7	this study

### (b) Recombinant DNA techniques

All oligonucleotides used for PCR are listed in table 5. Plasmid isolation, digestion with restriction endonucleases, site-directed mutagenesis by PCR and transformation into *E. coli* were performed by standard methods [29,30].

**Table 5.** Oligonucleotides used in this study.

oligonucleotide name	oligonucleotide sequence	template	plasmid created
CprK K133L F	CTTTAAAAACTACCTTACCAAAGTGGCTTATTATGCGCGAC	pMLK1	pMLK2
CprK K133L R	GTCGCGCATAATAAGCCACTTTGGTAAGGTAGTTTTTAAAG	pMLK1	pMLK2
CprK Y76F F	GCTTCTCTTTTATGCCGGCGGC	pMLK1	pMLK3
CprK Y76F R	GCCGCCGGCATAAAAGAGAAGC	pMLK1	pMLK3
CprK G85A F	GGCAATTCCCTGATCGCAAAATTATATCCTACG	pMLK1	pMLK4
CprK G85A R	CGTAGGATATAATTTTGCGATCAGGGAATTGCC	pMLK1	pMLK4
DB7 GFP F	GACTGGAAAGCAGATCTGCTCTGGG	*D. hafniense* DCB-2 genomic DNA	p-GFP_UV_-DB7
DB7 GFP R	GTGAAATTTATGATATCAAAAAAGTAG	*D. hafniense* DCB-2 genomic DNA	p-GFP_UV_-DB7

### (c) *In vivo* GFP_UV_-coupled activity assay

Overnight culture (150 μl) was plated onto 96-well optical bottom plates (Nunc). OCPA-related compounds (table 3) were then added to various end concentrations, and the plates placed on a rotary shaker at 37°C for 3 h. GFP fluorescence levels were measured using a Synergy HT microplate reader (BioTek) using a 400 nm, 30 nm bandpass excitation filter and a 508 nm, 20 nm bandpass emission filter. Data were collected using Gen5 v. 1.05 (Biotek) software. Fluorescence was normalized against cell density and measured by absorbance at 600 nm. The fluorescence ratio was plotted against effector concentration, and apparent *K*_d_ of binding was calculated using nonlinear curve fitting to a Hill plot.

**Figure 5.**
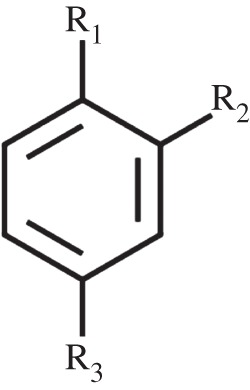
General structure of the effectors used in this study.

### (d) Western blot analysis of CprK soluble protein levels

To assess cellular levels of soluble CprK variants used in *in vivo* GFP-coupled activity assays, 1.5 ml of overnight culture was pelleted by centrifugation, and the supernatant was discarded. The pellet was resuspended with 300 μl BugBuster protein extraction reagent (Merck), supplemented with 10 μg ml^−1^ lysozyme, 10 μg ml^−1^ DNase and 10 μg ml^−1^ RNase. The mixture was incubated at 21°C, 300 r.p.m., for 10 min. The lysed cells were centrifuged at 10 000*g* 4°C, for 10 min. Soluble fractions were collected and concentrations determined using a Bradford assay (Sigma). The soluble fractions were added to SDS-loading buffer to a final amount of 30 μg protein. The proteins were then electro-transferred onto Immobilon PVDF membrane (Millipore), and detected using Universal His Western blotting kit 2 (Clontech).

### (e) p*K*_a_ calculations

Calculations of p*K*_a_ values were made with an implementation of the finite difference Poisson–Boltzmann method [31]. Relative dielectric values were assigned as 4 (protein) and 78.4 (water), with an ionic strength of 0.15 M, and amino acid model compound p*K*_a_ values as in previous work [31]. OCPA was assigned a p*K*_a_ of 8.5 and HPA 10.2. Energetics of pH-dependence were calculated according to Antosiewicz *et al*. [32]. The unbound dimer of CprK was used (3e5q) in binary complex with effector (3e5x), and ternary complex with effector and DNA (3e6c). In order to compare calculated K133 p*K*_a_ with that for the true unbound structure, a calculation was also made for the notional unbound form of CprK dimer extracted from the binary complex. The K133L mutant was modelled in Swiss PDB viewer [33].
